# Efficacy of fresh frozen plasma transfusion in decompensated cirrhosis patients with coagulopathy admitted to ICU: a retrospective cohort study from MIMIC-IV database

**DOI:** 10.1038/s41598-024-54379-0

**Published:** 2024-02-28

**Authors:** Xiangjie Fu, Danyang Yan, Wanting Huang, Xi Xie, Yiran Zhou, Huan Li, Yanjie Wang, Siya Pei, Run Yao, Ning Li

**Affiliations:** 1 Department of Blood Transfusion, Clinical Transfusion Research Center, National Clinical Research Center for Geriatric Disorders, Xiangya Hospital, Central South University, Changsha, Hunan China; 2 Department of Clinical Laboratory, Hunan Prevention and Treatment Institute for Occupational Diseases, University of South China, Changsha, Hunan China

**Keywords:** Decompensated cirrhosis, Coagulopathy, Fresh frozen plasma, Transfusion, Diseases, Medical research

## Abstract

We aimed to explore the association between FFP transfusion and outcomes of DC patients with significant coagulopathy. A total of 693 DC patients with significant coagulopathy were analyzed with 233 patients per group after propensity score matching (PSM). Patients who received FFP transfusion were matched with those receiving conventional therapy via PSM. Regression analysis showed FFP transfusion had no benefit in 30-day (HR: 1.08, 95% CI 0.83–1.4), 90-day (HR: 1.03, 95% CI 0.80–1.31) and in-hospital(HR: 1.30, 95% CI 0.90–1.89) mortality, associated with increased risk of liver failure (OR: 3.00, 95% CI 1.78–5.07), kidney failure (OR: 1.90, 95% CI 1.13–3.18), coagulation failure (OR: 2.55, 95% CI 1.52–4.27), respiratory failure (OR: 1.76, 95% CI 1.15–2.69), and circulatory failure (OR: 2.15, 95% CI 1.27–3.64), and even associated with prolonged the LOS ICU (β: 2.61, 95% CI 1.59–3.62) and LOS hospital (β: 6.59, 95% CI 2.62–10.57). In sensitivity analysis, multivariate analysis (HR: 1.09, 95%CI 0.86, 1.38), IPTW (HR: 1.11, 95%CI 0.95–1.29) and CAPS (HR: 1.09, 95% CI 0.86–1.38) showed FFP transfusion had no beneficial effect on the 30-day mortality. Smooth curve fitting demonstrated the risk of liver failure, kidney failure and circulatory failure increased by 3%, 2% and 2% respectively, for each 1 ml/kg increase in FFP transfusion. We found there was no significant difference of CLIF-SOFA and MELD score between the two group on day 0, 3, 7, 14. Compared with the conventional group, INR, APTT, and TBIL in the FFP transfusion group significantly increased, while PaO2/FiO2 significantly decreased within 14 days. In conclusion, FFP transfusion had no beneficial effect on the 30-day, 90-day, in-hospital mortality, was associated with prolonged the LOS ICU and LOS hospital, and the increased risk of liver failure, kidney failure, coagulation failure, respiratory failure and circulatory failure events. However, large, multi-center, randomized controlled trials, prospective cohort studies and external validation are still needed to verify the efficacy of FFP transfusion in the future.

## Introduction

Decompensated cirrhosis (DC), as an acute deterioration in liver function in a patient with cirrhosis, was usually accompanied by life-threatening bleeding and thrombosis due to hepatic synthetic dysfunction-induced coagulopathy^1,2^, with a 10–20% risk of in-hospital mortality^3,4^ and nearly 70% risk of 5-year mortality^5^. The approached to the management of DC patients with coagulopathy rely on targeted strategies aimed at preventing or treating hemorrhage and thrombotic complications^6^, thereby reducing the risk of mortality.

The presence of coagulopathy in critically ill patients, regardless of its etiology, increases the likelihood of hemorrhage by four to fivefold and is an independent risk factor for mortality^7^. How to manage patients with coagulopathy admitted to intensive care unit (ICU) due to liver disease is controversial^8^. American Association for the Study of Liver Diseases (AASLD) and American Gastroenterological Association (AGA) recommend against the routine use of fresh frozen plasma (FFP) for minor procedures such as paracentesis to correct an elevated PT/INR in a patient with liver disease^8–10^. Some studies, like Youssef et al., noted a possible failure of FFP transfusion to correct the coagulopathy in patients with liver disease^11^. Von Meijenfeldt et al., showed FFP transfusion resulted in increased thrombin generation in patients with liver disease, indicating a prothrombotic effect^12^. Nevertheless, a national multidisciplinary survey reveals significant heterogeneity of pre-procedural prophylactic FFP transfusion practices in patients with cirrhosis and discrepancies between guidelines and clinical practice. Especially for patients with cirrhosis and coagulopathy, many proceduralists still give FFP transfusion based on INR elevation to patients having low-risk procedures (30% to patients with an INR (1.6–1.9) and 46% to patients with an INR ≥ 2)^13,14^. A UK-based study, collecting 28-day data from 85 hospitals, revealed that 30% of cirrhosis patients received at least one blood component during their hospitalization, with FFP being prescribed in nearly 30% of these cases^15^. According to the Japanese guidelines, FFP transfusion is indicated to promote recovery from bleeding tendencies associated with coagulopathy or coagulation factor deficiency in liver disease^7^. And guidelines suggested FFP transfusion in cirrhosis patients with actively bleeding^16^. In addition, Liver Intensive Care Group of Europe (LICAGE) recommended that FFP should be administered in the setting of coagulopathy-associated clinically significant bleeding, while prophylactic use of FFP was not recommended^17^. But a retrospective study by Mohanty et al. reported that FFP transfusion was associated with significantly increased mortality at 42 days, failure to control bleeding at 5 days, and longer hospital stays in patients with cirrhosis and acute variceal hemorrhage^18^.

The efficacy of FFP in the treatment of DC patients with coagulopathy, whether characterized by a high risk of bleeding or active bleeding, remains controversial. Additionally, there have been limited studies exploring the effects of FFP transfusion on mortality or other adverse outcomes in DC patients with coagulopathy. Therefore, the primary aim of our study was to investigate the effect of FFP transfusion on mortality in DC patients with coagulopathy and to further understand the effect of FFP transfusion on organ failure (liver failure, kidney failure, coagulation failure, respiratory failure, and circulatory failure). This retrospective study will provide evidence-based insights into the controversial topic of FFP transfusion in DC patients with coagulopathy, and provide a literature reference for clinical practice to improve patient outcomes.

## Materials and methods

### Study design

This study conducted a retrospective cohort analysis utilizing the Medical Information Mart for Intensive Care IV (MIMIC-IV version 2.0) database. The MIMIC-IV database, an extensive open-source medical record repository, comprises data from 382,278 individuals admitted to the Intensive Care Unit (ICU) of Beth Israel Deaconess Medical Center (BIDMC) between 2008 and 2019. The database primarily includes patient demographics, laboratory test results, vital sign measurements, and prescriptions documented by healthcare professionals. To obtain access to the database, we completed the course “Protecting Human Research Participants” on the website of the National Institutes of Health and obtained certification (Record ID:40060500). The project was approved by the institutional review boards of Massachusetts Institute of Technology (MIT) and BIDMC and was granted a waiver of informed consent.

### Patient population

All patients diagnosed with DC were enrolled in the study. DC was determined based on the assessment of clinical, laboratory, imaging data, endoscopic and histological findings^19^. DC is characterized by jaundice, ascites, hepatic encephalopathy, hepatorenal syndrome or variceal hemorrhage, and it represents an acute deterioration in liver function occurring in a patient with cirrhosis^4,20^. The inclusion criteria were: (1) significant coagulopathy (INR > 1.8 and/or platelet count < 50 × 10^9^/L)^21^; (2) aged exceed 18 years. Exclusion criteria were: (1) duplicated ICU admission records; (2) diagnosis with pregnancy, advanced malignancy, or surgical emergencies; (3) diagnosis with coagulopathy disorders other than liver disease; (4) diagnosis with recipient of liver transplantation or end-stage renal disease^22^. Patients who received FFP transfusion during their ICU stay were categorized into the FFP transfusion group, while the remaining patients constituted the conventional group. Decision on FFP transfusion was based on the clinical judgment of the attending physician, and dosage was determined considering the weight of patients. All patients received standard and conventional treatments, including albumin supplementation, coagulation correction, liver protection, antiviral treatment, essential anti-infective treatment, management of associated complications and etc.

### Data collection

Structured Query Language (SQL) and PostgreSQL tools (version 9.6) were used to extract the data information of patients with demographic data, score system data, laboratory data, anticoagulants use, bleeding and comorbidities. The demographic data included age and gender. Score system included the Model for End-Stage Liver Disease (MELD) score and Chronic Liver Failure-Sequential Organ Failure Assessment (CLIF-SOFA) score. The laboratory examination data included platelet (PLT), alanine aminotransferase (ALT), aspartate transaminase (AST), PT, APTT and INR. Bleeding evens is characterized by (1) hemoglobin (Hb) drop ≥ 2 g/dL or ≥ 2 units of packed red blood cell transfusion required because of bleeding; (2) Symptomatic critical site bleeding (central nervous system, retroperitoneal, pericardial, ophthalmologic, intra-articular)^23^. Comorbidities included hypertension, diabetes, chronic obstructive pulmonary disease (COPD) and renal disease.

We defined the day of the first FFP transfusion as day 0 in the FFP transfusion group, and the day of admission to ICU as day 0 in the conventional group. CLIF-SOFA score and MELD score were recorded at indicated time (0, 3, 7, and 14 day). Laboratory indexes for both groups were recorded from day 0 to day 14.

### Definition and clinical outcomes

MELD score is suited to short-term prognostication for patients with decompensated cirrhosis and it is used to prioritize organ allocation on the transplant waitlist^24^. MELD score is calculated using serum levels of bilirubin, creatinine, INR and etiology of the underlying liver disease. The calculation formula is as follows: MELD score = 0.378ln [bilirubin (mg/dL)] + 1.12ln (INR) + 0.95ln [creatinine (mg/dL)] + 0.64 (etiology: cholestatic or alcoholic 0, other 1). CLIF-SOFA score includes sub-scores ranging from 0 to 4 for each of 6 components (liver, kidneys, brain, coagulation, circulation and lungs), with higher scores indicating more severe organ impairment^25^. Definitions of Organ Failures are as follows: kidney failure was defined by a serum creatinine ≥ 2 mg/dL or the use of renal replacement. Liver failure was defined by a serum bilirubin ≥ 12 mg/dL. Coagulation failure was defined by an INR > 2.5 and/or a platelet count ≤ 20 × 10^9^/L. Cerebral failure was defined by grade III or IV hepatic encephalopathy. Respiratory failure was defined by a ratio of partial pressure of arterial oxygen to fraction of inspired oxygen (FiO_2_) of ≤ 200 or a ratio of pulse oximetric saturation (SpO_2_) to FiO_2_ of ≤ 200. Circulatory failure was defined by the use of dopamine, dobutamine or terlipressin.

The primary outcome was mortality including 30-day, 90-day and in-hospital mortality. The secondary outcomes were length of ICU stay (LOS ICU), length of hospital stay (LOS hospital) and organ failures.

### Statistical analysis

Continuous variables were shown as mean with standard deviations or median with interquartile range (Q1-Q3), and tested by Student’s t-test (normal distribution) or Mann–Whitney U-test (non-normal distribution). Categorical variables were presented as numbers (n) with percentages (%), and tested by chi-square test or Fisher’s exact test.

Propensity score matching (PSM) was used to adjust for differences in baseline variables, including age, gender, MELD score, CLIF-SOFA score, PLT, AST, ALT, PT, APTT, INR, anticoagulants use, bleeding, hypertension, diabetes, COPD and renal disease^26^. One-to-one nearest neighbor matching with a caliper width of 0.05 was applied in the present study. Logistic regression was used to assess the odds ratios (OR) and 95% confidence intervals (CIs) for liver failure, kidney failure, coagulation failure, respiratory failure and circulatory failure risks. Patients with pre-existing organ failure at admission were excluded, the remaining patients without organ failure at admission were included in this logistic analysis, and new onset organ failure after admission was included as an outcome variable in this logistic regression analysis. Cox regression was used to estimate the hazard ratios (HR) and 95% CIs for 30-day, 90-days and in-hospital mortality risks. Linear regression was employed to examine the β and 95% CIs for LOS ICU and LOS hospital. Smooth curve fitting was performed to determine the association between FFP transfusion volume and the risk of organ failure. In order to ensure the robustness of our finding, PSM, univariate analysis, multivariable analysis, propensity score-based inverse probability of treatment weighing (IPTW) and covariate adjustment using propensity score (CAPS) were implemented in the sensitivity analysis. The longitudinal data were laboratory indexes over time, with repeated measurements from 0 to 14 days. Generalized additive mixed model (GAMM) was used to analyze the association between the early change of these indexes and treatment modalities. All statistical analyses were involved in the study using the R software package (http://www.R-project.org, The R Foundation for Statistical Computing, version 3.4.3) and Statistics software (EmpowerStats, version 4.1). A two-sided *P* < 0.05 was considered statistically significant.

### Ethical consideration

The studies involving human participants were reviewed and approved by the institutional review boards of the Massachusetts Institute of Technology and Beth Israel Deaconess Medical Center. Informed consent was waived in accordance with the national legislation and the institutional requirements.

## Results

### Participants selection

**Figure 1:**
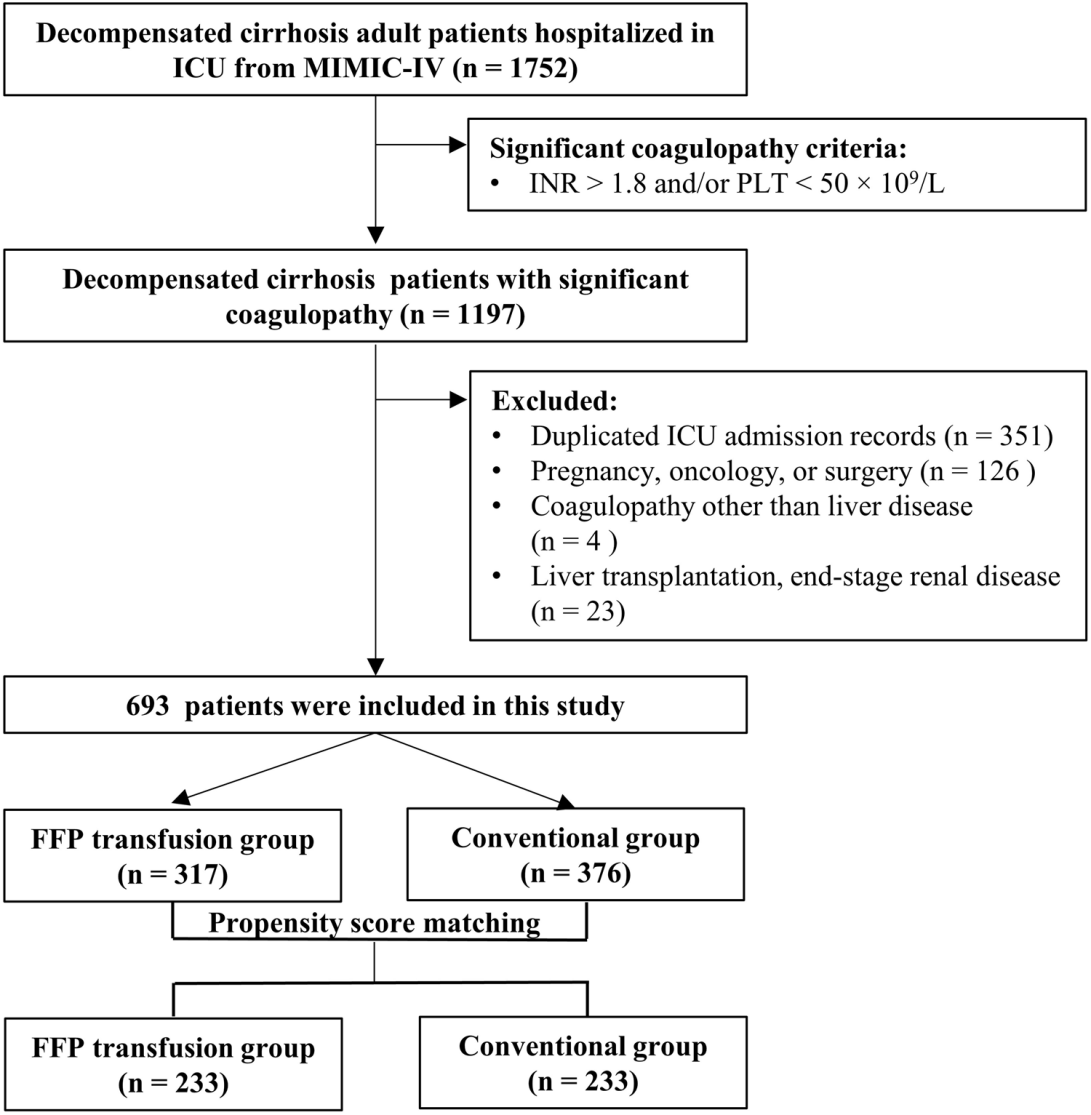
Flow chart of the study.

A total of 1752 patients with DC hospitalized in ICU were identified from MIMIC-IV database. Among them, a total of 1197 patients met the criteria for significant coagulopathy. 504 patients were excluded for the following reasons: 351 patients with duplicated ICU admission records, 126 patients with pregnancy, oncology, or surgery, 4 patients with purpura, and 23 patients with liver transplantation or end-stage renal disease. The remaining 693 patients were enrolled in this study, with 317 cases in the FFP transfusion group and 376 cases in the conventional group. After PSM, there were eventually 233 cases in each group. (Fig. 1).

### Baseline characteristics of DC cohort before and after propensity score matching (PSM)

The baseline demographic and clinical characteristics of DC cohort in the FFP transfusion group and conventional group were presented in Table 1. Before matching, there were significantly differences in age, MELD score, CLIF-SOFA score, laboratory data (PLT, AST, ALT, PT, APTT, and INR), anticoagulants use and bleeding between two groups (P < 0.05). After matching, there was no statistical difference in the baseline demographic and clinical characteristics between two groups (P > 0.05).

**Table 1:** The baseline characteristics of DC patients with significant coagulopathy in the FFP transfusion group and conventional group before and after PSM.

Variable	Before match			After match		
	FFP transfusion group (n = 317)	Conventional group (n = 376)	*P*-value	FFP transfusion group (n = 233)	Conventional group (n = 233)	*P*-value
Demographic data						
Age (year)	56.03 ± 10.78	59.51 ± 11.63	< 0.01*	56.29 ± 10.86	57.03 ± 10.66	0.46
Gender			0.22			1.00
Male	200 (63.09%)	220 (58.51%)		145 (62.23%)	145 (62.23%)	
Female	117 (36.91%)	156 (41.49%)		88 (37.77%)	88 (37.77%)	
Score system						
MELD score	33.00 (27.00–40.00)	28.00 (22.75–35.00)	< 0.01*	32.00 (26.00–38.00)	31.00 (26.00–38.00)	0.49
CLIF-SOFA score	13.00 (10.00–15.00)	10.00 (7.00–13.00)	< 0.01*	12.00 (10.00–15.00)	11.00 (8.00–14.00)	0.07
Laboratory data						
PLT (10^9^/L)	56.00 (40.00–85.00)	66.00(40.50–108.50)	< 0.01*	61.00 (42.00–96.00)	64.00 (40.00–101.00)	0.83
AST (U/L)	89.5 (48.00–343.50)	83.0 (43.00–146.00)	< 0.01*	85.0 (47.00–308.00)	91.0 (46.00–177.00)	0.47
ALT (U/L)	41.0 (23.00–102.75)	37.5 (21.00–66.25)	0.03*	40.0 (22.00–102.00)	40.0 (23.00–77.00)	0.89
PT (sec)	27.40 (23.00–35.10)	24.10 (20.70–30.98)	< 0.01*	26.70 (22.40–32.90)	26.00 (21.70–33.00)	0.21
APTT (sec)	54.95 (44.62–79.97)	48.35 (38.98–67.12)	< 0.01*	53.70 (43.10–77.30)	51.40 (41.40–70.60)	0.07
INR	2.60 (2.20–3.30)	2.30 (1.90–2.90)	< 0.01*	2.50 (2.10–3.30)	2.40 (2.00–3.10)	0.15
Anticoagulants use	292 (92.11%)	312 (82.98%)	< 0.01*	211 (90.56%)	205 (87.98%)	0.37
Bleeding	196 (61.83%)	153 (40.69%)	< 0.01*	128 (54.94%)	113 (48.50%)	0.16
Comorbidities						
Hypertension	126 (39.75%)	176 (46.81%)	0.06	94 (40.34%)	99 (42.49%)	0.64
Diabetes	85 (26.81%)	108 (28.72%)	0.58	60 (25.75%)	63 (27.04%)	0.75
CPOD	61 (19.24%)	81 (21.54%)	0.46	48 (20.60%)	50 (21.46%)	0.82
Renal disease	68 (21.45%)	97 (25.80%)	0.18	51 (21.89%)	53 (22.75%)	0.82

Results are expressed as mean ± SD, median (Q1-Q3), or n (%). *P < 0.05. FFP, fresh frozen plasma; MELD, the model for end-stage liver disease score; SOFA, the sequential organ failure assessment score; PLT, platelet; AST, aspartate aminotransferase; ALT, alanine aminotransferase; PT, prothrombin time; APTT, activated partial thromboplastin time; INR, international normalization ratio; COPD, chronic obstructive pulmonary disease.

### Association between FFP transfusion and primary and secondary outcomes

In post-matched cohort, the 30-day, 90-day, and in-hospital mortality rates were 51.07%, 56.65% and 44.64% in FFP transfusion group, respectively and 46.35%, 54.51% and 38.20% in conventional group, respectively, and there were no statistically significant differences between two groups. The result of Cox logistic regression showed that there was no significant increase in mortality risk of 30-day (HR: 1.08, 95% CI 0.83–1.40), 90-day (HR: 1.03, 95% CI 0.80–1.31), and in-hospital mortality (HR: 1.30, 95% CI 0.90–1.89) (Table 2).

**Table 2:** Clinical outcomes analysis with propensity score-matched cohort.

Outcomes	Conventional group (n = 233)	FFP transfusion group (n = 233)	*P*-value	HR/OR/β (95% CI)	*P*-value
Primary outcomes					
30-day mortality, n/N (%) ^a^	108/233 (46.35%)	119/233 (51.07%)	0.308	1.08 (0.83, 1.40)	0.58
90-day mortality, n/N (%) ^a^	127/233 (54.51%)	132/233 (56.65%)	0.641	1.03 (0.80, 1.31)	0.84
In-hospital mortality, n/N (%)^a^	89/233 (38.20%)	104/233 (44.64%)	0.158	1.30 (0.90, 1.89)	0.16
Secondary outcomes					
Liver failure, n/N (%)^b^	26/156 (16.67%)	63/168 (37.50%)	< 0.01*	3.00 (1.78, 5.07)	< 0.01*
Kidney failure, n/N (%)^b^	38/109 (34.86%)	69/137 (50.36%)	0.02*	1.90 (1.13, 3.18)	0.02*
Coagulation failure, n/N (%)^b^	44/114 (38.60%)	80/130 (61.54%)	< 0.01*	2.55 (1.52, 4.27)	< 0.01*
Respiratory failure, n/N (%)^b^	55/193 (28.50%)	77/187 (41.18%)	< 0.01*	1.76 (1.15, 2.69)	< 0.01*
Circulatory failure, n/N (%)^b^	32/122 (26.23%)	58/144 (43.28%)	< 0.01*	2.15 (1.27, 3.64)	< 0.01*
LOS ICU (day), [median (IOR)]^c^	2.35 (1.09–5.12)	4.16 (2.35–8.96)	< 0.01*	2.61 (1.59, 3.62)	< 0.01*
LOS hospital (day), [median (IOR)]^c^	11.29 (4.21–21.79)	15.59 (8.79–29.60)	< 0.01*	6.59 (2.62, 10.57)	< 0.01*

Results are expressed as mean ± SD, median (Q1-Q3), or n (%). *P < 0.05. HR, hazard ratio; OR, odds ratio; CI, confidence interval; Ref, reference; LOS ICU, length of ICU stay; LOS hospital, length of hospital stay.

^a^The effect values in COx regression analysis are represented as HR.

^b^The effect values in logistic regression analysis are represented as OR.

^c^The effect values in linear regression analysis are represented as β.

**Figure 2:**
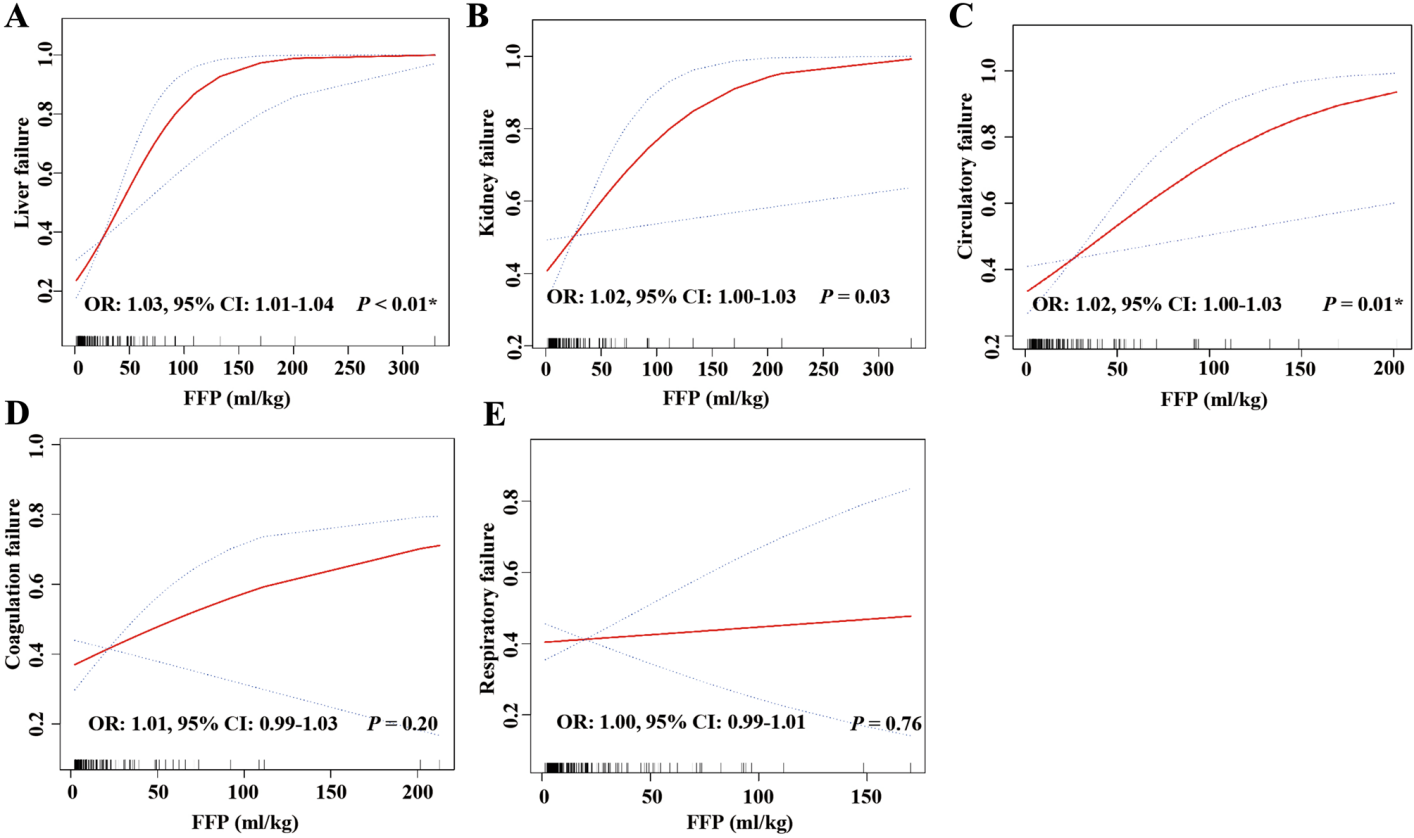
Smoothing spline fitting curve. (**A**) liver failure; (**B**) kidney failure; (**C**) circulatory failure; (**D**) coagulation failure; (**E**) respiratory failure. The linear plots are displayed with red dotted lines, and the blue dotted lines represent 95% CI. OR, odds ratio; CI, confidence interval. *P < 0.05.

The results of logistic regression analysis showed FFP transfusion was associated with increased risk of liver failure (OR: 3.00, 95% CI 1.78–5.07), kidney failure (OR: 1.90, 95% CI 1.13–3.18), coagulation failure (OR: 2.55, 95% CI 1.52–4.27), respiratory failure (OR: 1.76, 95% CI 1.15–2.69), and circulatory failure (OR: 2.15, 95% CI 1.27–3.64) (Table 2). We applied smoothing spline fitting to visualize the association between the FFP transfusion volume and risk of organ failure in DC patients with coagulopathy. The risk of liver failure, kidney failure and circulatory failure increased by 3% (OR: 1.03, 95% CI 1.01–1.04), 2% (OR: 1.02, 95% CI 1.00–1.03) and 2% (OR: 1.02, 95% CI 1.00–1.03) respectively, for each 1 ml/kg increase in FFP transfusion (Fig. 2A–C). While the FFP transfusion volume was no significantly associated with the risk of coagulation failure (OR: 1.01, 95% CI 0.99–1.03) and respiratory failure (OR: 1.00, 95% CI 0.99–1.01) (Fig. 2D,E). Moreover, we found that the length of ICU stays [4.16 (2.35–8.96) vs 2.35 (1.09–5.12)] and hospital stays [15.59 (8.79–29.60) vs 11.29 (4.21–21.79)] were longer in the FFP transfusion group than the conventional group (P < 0.01) (Table 2).

**Figure 3:**
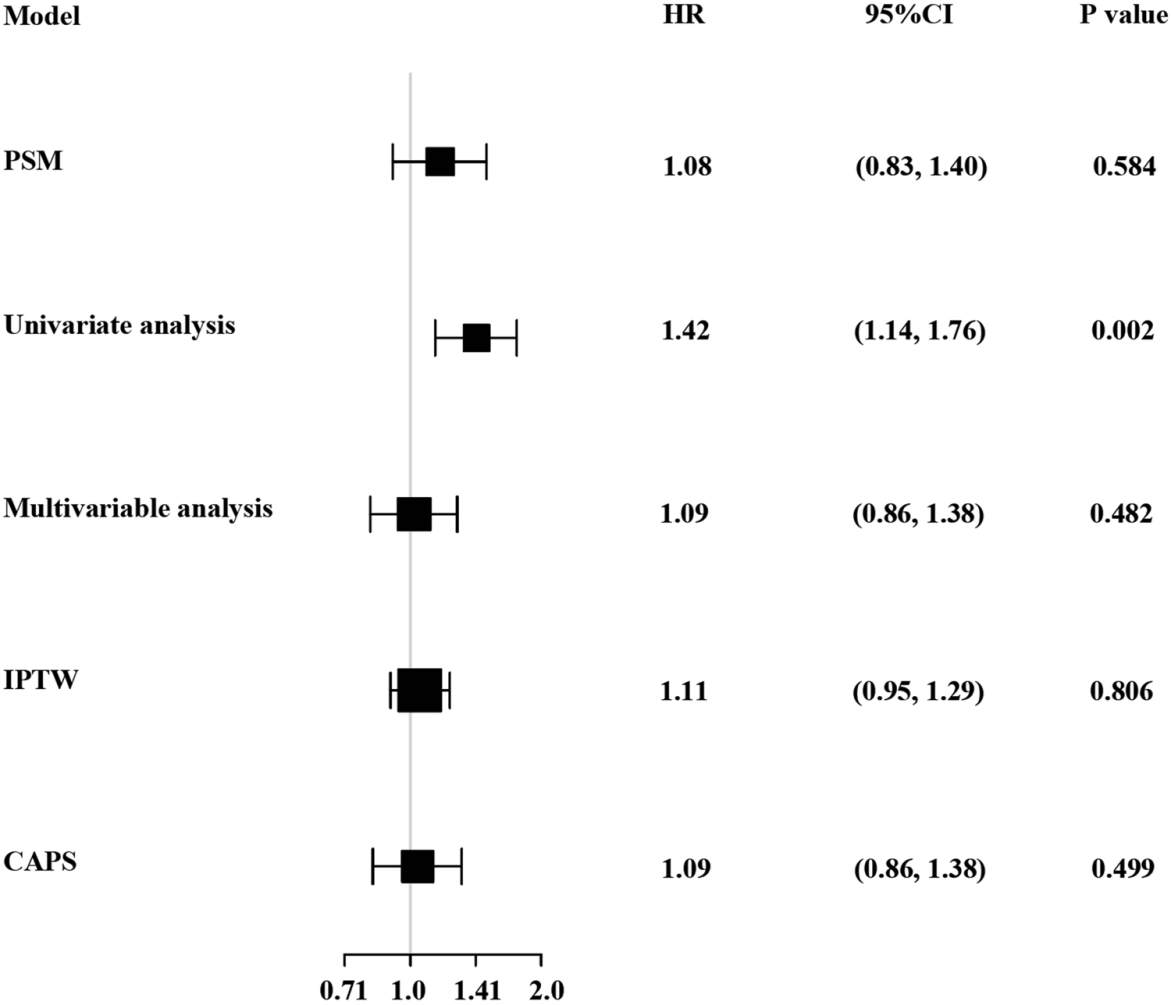
Association between FFP transfusion and 30-day mortality in different models. The HR (hazard ratios) and 95% CI (confidence intervals) were calculated dependent on the method of PSM (propensity score matching), univariate COX regression model (adjusted for none), multivariate COX regression model (adjusted for age, gender, MELD score, CLIF-SOFA score, PLT, AST, ALT, PT, APTT, anticoagulants use, bleeding), IPTW (inverse probability of treatment weighted), and CAPS (covariate adjustment using propensity score).

Sensitivity analysis demonstrated that FFP transfusion had no beneficial effect on the mortality risk of 30-day whether utilizing the method of Multivariable analysis (HR: 1.09, 95% CI 0.86, 1.38), IPTW (HR: 1.11, 95% CI 0.95–1.29) or CAPS (HR: 1.09, 95% CI 0.86–1.38) (Fig. 3).

### Early changes of CLIF-SOFA score, MELD score in two groups of PSM cohort

**Figure 4:**
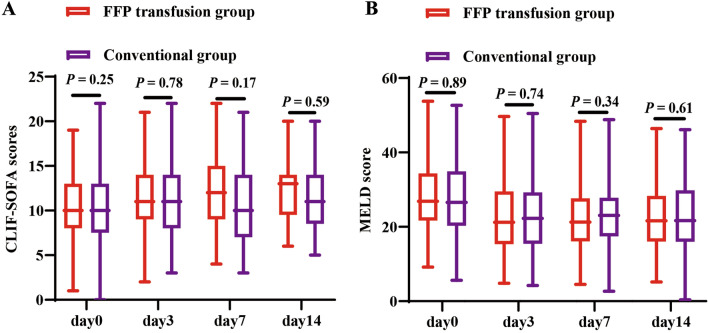
The change of grade CLIF-SOFA (**A**) and MELD scores (**B**) in DC patients with significant coagulopathy.

The changes in CLIF-SOFA score and MELD score at 0, 3, 7, 14 days between the FFP transfusion group and the conventional group were visualized in Fig. 4. There was no significant difference of CLIF-SOFA score between the two group on day 0 (10.58 vs. 10.17, P = 0.25), day 3 (11.40 vs. 11.28, P = 0.78), day 7 (11.93 vs. 10.95, P = 0.17), and day 14 (12.14 vs. 11.53, P = 0.59). The MELD scores between the FFP transfusion group and the conventional group on day 0 (27.92 vs. 27.79, P = 0.89), day 3 (22.54 vs. 22.89, P = 0.74), day 7 (22.39 vs. 23.57, P = 0.34), and day 14 (22.66 vs. 23.42, P = 0.61) were no significant difference.

### Association between early changes of laboratory indexes and treatment modalities in PSM cohort

**Figure 5:**
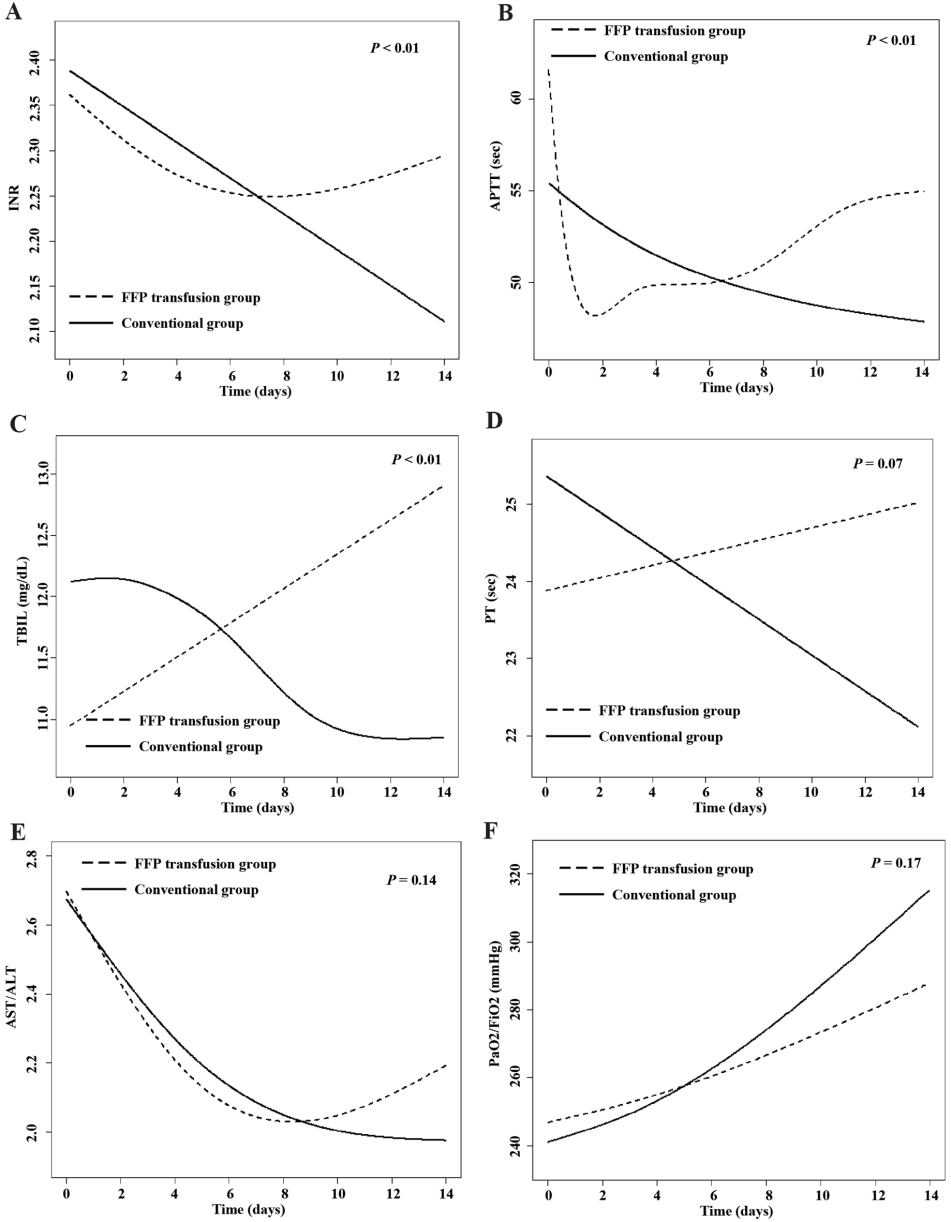
The dynamic changes of laboratory indexes within 14 days in DC patients with significant coagulopathy, using the generalized additive mixed model. (**A**) INR; (**B**) APTT; (**C**) TBIL; (**D**) PT; (**E**) AST/ALT; (**F**) PaO_2_/FiO_2_.

The changes in laboratory indexes over time between the FFP transfusion group and the conventional group were observed in Fig. 5 and Table 3. The conventional group experienced an average decrease of 0.02/day (95% CI − 0.03 to − 0.01), 0.58 s/day (95% CI − 0.78 to − 0.38), 0.06/day (95% CI − 0.07 to − 0.05), 0.12 mg/dL/day (95% CI − 0.17 to − 0.07) in INR, APTT, AST/ALT, TBIL, respectively, and an average increase of 4.73/day (95% CI 2.49–6.97) in PaO_2_/FiO_2_. In contrast, compared with the conventional group, the FFP transfusion group demonstrated an additional increase of 0.01 (95% CI 0.004–0.02)/day in INR (Fig. 5A, and Table 3), 0.61 (95% CI 0.36–0.87) sec/day in APTT (Fig. 5B, and Table 3) and 0.26 (95% CI 0.20–0.33) mg/dL/day in TBIL (Fig. 5C, and Table 3), respectively. While the FFP transfusion group showed no statistically difference in PT, AST/ALT and PaO_2_/FiO_2_ compared with the conventional group over time (Fig. 5D–F, and Table 3).

**Table 3:** Association between early changes in laboratory indexes and treatment modalities in DC patients with significant coagulation.

Indexes	FFP transfusion	Day	Day × FFP transfusion
PT (sec)	− 2.38 (− 4.62, − 0.14)*	− 0.22 (− 0.47, 0.03)	0.30 (− 0.02, 0.63)
INR	− 0.16 (− 0.31, 0.01)*	− 0.02 (− 0.03, − 0.01)*	0.01 (0.004, 0.02)*
APTT (sec)	− 4.13(− 7.13, − 1.13)*	− 0.58 (− 0.78, − 0.38)*	0.61 (0.36, 0.87)*
AST/ALT	0.07 (− 0.16, 0.29)	− 0.06 (− 0.07, − 0.05)*	0.01 (− 0.004, 0.03)
TBIL (mg/dL)	− 0.44 (− 2.55, 1.66)	− 0.12 (− 0.17, − 0.07)*	0.26 (0.20, 0.33)*
PaO2/FiO2	13.3 (− 12.1, 38.7)	4.73 (2.49, 6.97)*	− 1.88 (− 4.54, 0.79)

Results were expressed as β (95% CI). *P < 0.05. CI, confidence interval. FFP Transfusion indicated the difference of indexes at day 0 in FFP transfusion group compared with conventional group. Day indicated the mean of the increasing of indexes daily in conventional group. Day × FFP Transfusion indicated the average increasing of indexes daily in FFP transfusion compared with conventional group.

## Discussion

In this retrospective cohort study, we investigated the efficacy of FFP transfusion in DC patients with significant coagulopathy admitted to ICU. Our findings manifested that FFP transfusion had no beneficial effect on the 30-day, 90-day, in-hospital mortality, and was even associated with prolonged the LOS ICU and LOS hospital in DC patients with significant coagulopathy. Mohanty et al.^18^ showed FFP transfusion was independently associated with increased the risk of 42-day mortality and the length of hospital stay in patients with cirrhosis and acute variceal hemorrhage. A study reported FFP transfusion had a negative impact on the overall survival in patients with colorectal liver metastases^27^. These findings were similar to those of our study. Similar effects of FFP transfusion have been reported in other diseases. A retrospective single-center cohort by Dietrich et al.^28^ and a retrospective cohort study by Qin et al.^29^ both revealed FFP transfusion had no beneficial influence on 30-day and 90-day mortality in patients with sepsis or septic shock. A prospective observational study of critically ill patients showed no significant difference in the median LOS hospital and mortality between prophylactic FFP group and no-FFP group^30^.

We also found FFP transfusion was associated with increased risk of liver failure, kidney failure, coagulation failure, respiratory failure, and circulatory failure events. And there was positive association between FFP transfusion volume and liver failure, kidney failure as well as respiratory failure in DC patients with significant coagulopathy. Although FFP contains a variety of coagulation factors and is a classic strategy for reversing anticoagulation, FFP transfusion in patients with cirrhosis and coagulopathy may have inherent risks (e.g. prothrombotic effects, transfusion-related complications) without conferring clear benefits (e.g. only slightly increased thrombin generation)^31^. Recent literature indicated the large volume of FFP can be detrimental as blood volume expansion may exacerbate circulatory overload and thereby worsen portal hypertension^18,32^. In a nationwide cohort study, Xu et al.^33^ found that a higher volume of FFP transfusion was associated with increased risks of in-hospital mortality and postoperative complications in patients undergoing surgery without massive transfusion. Dettmer et al.^34^ demonstrated a higher volume of FFP was associated with in-hospital mortality in patients managed for large volume hemorrhage in a medical ICU. In addition, FFP transfusion could cause transfusion-related complications, including transfusion-associated acute lung injury, transfusion-associated circulatory overload and multi-organ failure^28,35^, which might be one of explanations for the positive association between FFP transfusion and the risk of liver failure and respiratory failure in this study.

Furthermore, we explored changes in CLIF-SOFA score and MELD score between two groups, we found no significant difference in CLIF-SOFA score and MELD score between the two groups on day 0, 3, 7, 14. The CLIF-SOFA and MELD score reflected multiorgan dysfunction and predicted the risk of mortality in patients with cirrhosis^36,37^. We further observed the changes in indexes (PT, INR, APTT, ALT/AST, TBIL and PaO_2_/FiO_2_) over time between the FFP transfusion group and the conventional group. TBIL showed an increased tendency in FFP transfusion group compared to conventional group in this study, which supported that FFP transfusion failed to improve liver function. In addition, our results showed a tendency for prolongation of coagulation indexes (INR and APTT) in the FFP group compared to the conventional group, which suggested that FFP transfusion didn’t correct a prolonged coagulation indexes in DC patients with significant coagulopathy. These results consistently supported evidence that there was no documented benefit of FFP transfusion in DC patients with significant coagulopathy. Islam et al.^38^ also reported that coagulopathy in liver cirrhosis was a complicated issue, and current coagulation tests and FFP transfusion could not provide suitable strategies for predicting and preventing bleeding episodes. Guidelines indicated that FFP transfusion was inappropriate to correct prolonged INR in the absence of bleeding in advanced liver disease^39,40^.

For patients with active bleeding in patients with cirrhosis, despite the lack of conclusive evidence of efficacy, guidelines do suggest the policy of FFP administration^16,17^. To conduct a more comprehensive investigation, we performed subgroup analyses and found that FFP transfusion had no benefit in 30-day mortality risk across non-bleeding (HR: 1.28, 95% CI 0.95–1.71) and bleeding (HR: 2.07, 95% CI 1.45–2.95) subgroups (Supplementary Table 1). Furthermore, we assessed whether FFP transfusion had impact on coagulation parameters and blood transfusion volumes in the bleeding subgroup. As showed in Supplementary Table 2, we found that the RBC and PLT transfusion volume was higher in the FFP transfusion group than the conventional group. While the Cryoprecipitate transfusion volume had no significant difference in the FFP transfusion group than the conventional group. In terms of hemostatic effect, the results indicated that, compared with the conventional group, the FFP transfusion group exhibited increased trend in HB, APTT, PT and INR from day 0 to day 14, but the differences were not statistically significant (Supplementary Fig. 1). The above results indicated that FFP had no significant effect on the improvement of hemostasis, anemia, and coagulation in patients experiencing bleeding. Consistantly, FFP transfusion had no beneficial effect on DC patients with coagulopathy, whether characterized by a high risk of bleeding or active bleeding. These data suggest a widespread belief that FFP transfusion should be restrictive, but is discordant with the findings of a seris of survey, which was performed in the same health care system from which we surveyed clinicians’ views.

There were still some limitations in this study. Firstly, this was a retrospective study, and bias was inevitable. We employed propensity score matching to adjust for confounding factors as much as possible in the statistical analysis to control the potential bias. As a case–control study, many confounders for matching may lead to bias in the outcome, we used strong confounders from literature to minimize bias. Secondly, we excluded patients with pregnancy, oncology, surgery, purpura, liver transplantation and end-stage renal disease. Therefore, the findings of this study might not be generalizable to these specific patient groups. And in this study, the selection matched was biased by the degree of complexity of the patients. We performed propensity score (PSM) matching to ensure, as much as possible, the comparability of the two patient groups in terms of disease severity, coagulation status and bleeding status. We conducted sensitivity analysis to further enhance the credibility and stability of our results. Thirdly, as an observational study, this study was only able to draw association conclusions, and no causal relationship between FFP transfusion and the prognosis of DC patients with significant coagulopathy could be obtained. Further prospective, randomized controlled studies were warranted to validate and strengthen our findings. Finally, the physician's decision to administer FFP without adhering to a specific protocol may rely solely on their clinical judgment, potentially influenced by laboratory tests such as aPTT and INR ratios. However, it's worth noting that these parameters may not be the most reliable indicators for guiding FFP transfusions.

## Conclusions

FFP transfusion had no beneficial effect on the 30-day, 90-day, in-hospital mortality, and was even associated with prolonged the LOS ICU and LOS hospital in DC patients with significant coagulopathy. FFP transfusion was associated with increased risk of liver failure, coagulation failure and respiratory failure events. And there was positive association between FFP transfusion volume and liver failure as well as respiratory failure in DC patients with significant coagulopathy. Future randomized studies aimed to validate the causal relationship between FFP transfusion and the prognosis of DC patients with significant coagulopathy were warranted. So, physicians should choose a more rational approach to FFP transfusion practice in DC patients with coagulopathy.

### Supplementary Information

Supplementary Information.

## Data Availability

The data used for this study, though not available in a public repository, will be made available to other researchers upon reasonable request. Please contact the corresponding authors Run Yao (yaorunxy@csu.edu.cn) or Ning Li (liningxy@csu.edu.cn) via E-mail.
