# Supplementary material for: Efficacy of fresh frozen plasma transfusion in decompensated cirrhosis patients with coagulopathy admitted to ICU: a retrospective cohort study from MIMIC-IV database
Source: Sci Rep. 2024 Feb 28;14:4925. doi: 10.1038/s41598-024-54379-0 (PMC10902319; doi:10.1038/s41598-024-54379-0)
Supplement: Supplementary file 1 — Supplementary Information. [file 41598_2024_54379_MOESM1_ESM.docx]

**Supplementary Results**

Supplementary Table 1. Subset analysis for DC patients with significant coagulopathy before PSM.

| **Subgroups** | **N** | **Conventional group, n (%)** | **FFP transfusion group, n (%)** | **30-day mortality** | | | |
| --- | --- | --- | --- | --- | --- | --- | --- |
|  |  |  |  | **HR** | **95% CI** | **P-value** | ***P* for interaction** |
| **Bleeding** |  |  |  |  |  |  | 0.040* |
| NO | 344 | 114 (51.12%) | 75 (61.98%) | 1.28 | 0.95, 1.71 | 0.101 |  |
| YES | 349 | 44 (28.76%) | 101 (51.53%) | 2.07 | 1.45, 2.95 | <0.01* |  |

Supplementary Table 2. Total blood product volume of DC patients with significant coagulopathy and bleeding in the FFP transfusion group and conventional group after PSM

| **Variable** | **FFP transfusion**  **group (n=128)** | **conventional**  **group (n=113)** | **P-value** |
| --- | --- | --- | --- |
| RBC transfusion (mL), [median (IQR)] | 1384.00 (350.00-3164.00) | 700.00 (0.00-1775.00) | 0.005* |
| Cryoprecipitate transfusion (mL), [median (IQR)] | 0.00 (0.00-242.75) | 0.00 (0.00-196.00) | 0.064 |
| PLT transfusion (mL), [median (IQR)] | 279.50 (0.00-873.25) | 0.00 (0.00-470.00) | 0.008* |


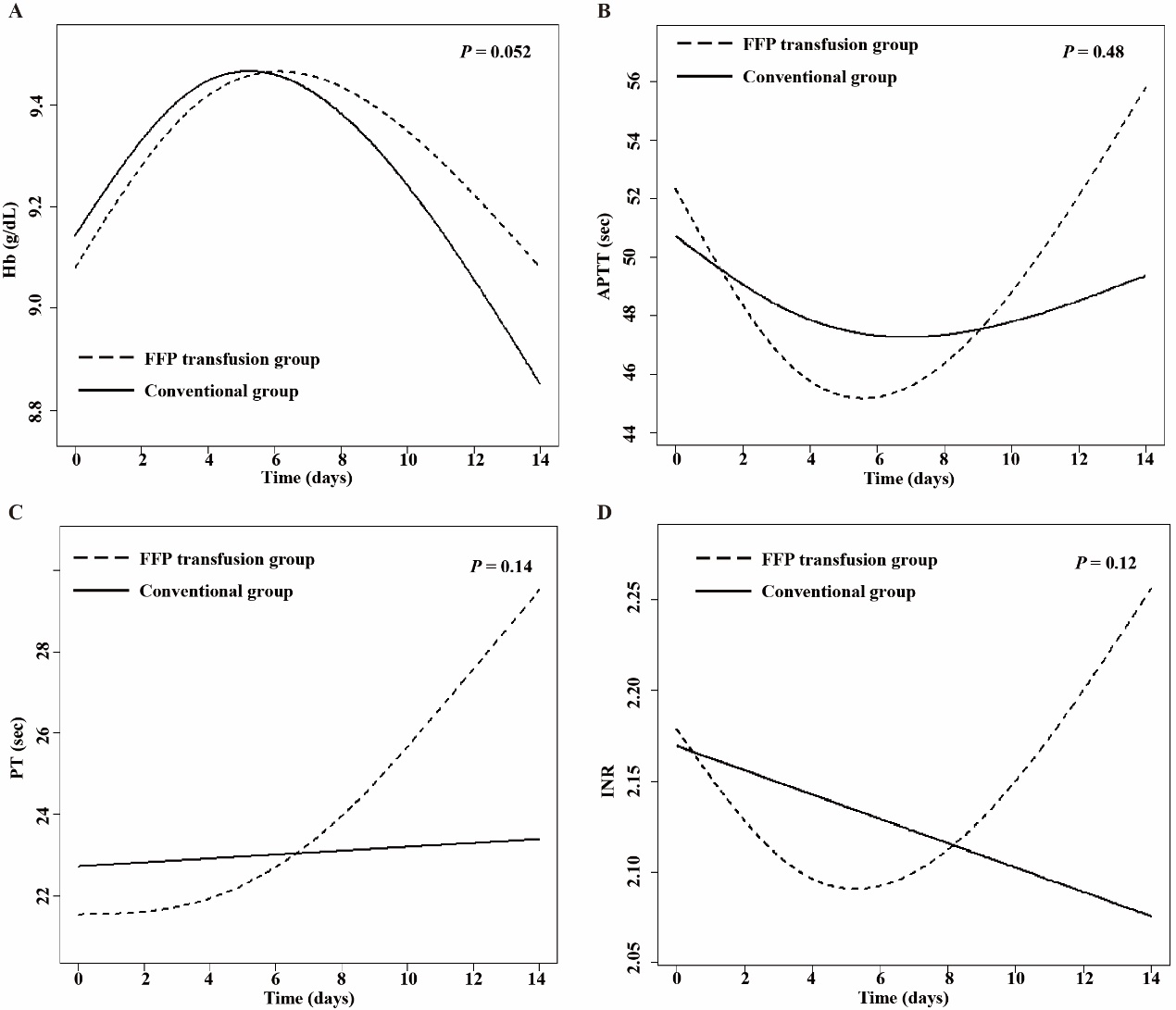


Supplementary Figure 1. The dynamic changes of laboratory indexes within 14 days in DC patients with significant coagulopathy and bleeding, using the generalized additive mixed model. (A) Hb; (B) APTT; (C) PT; (D) INR.
